# The mouse olfactory peduncle. 3. Development of neurons, glia, and centrifugal afferents

**DOI:** 10.3389/fnana.2014.00044

**Published:** 2014-06-05

**Authors:** Peter C. Brunjes, Lindsay N. Collins, Stephen K. Osterberg, Adriana M. Phillips

**Affiliations:** Department of Psychology, University of Virginia, CharlottesvilleVA, USA

**Keywords:** olfactory cortex, anterior olfactory nucleus, cortical interneurons, cholinergic afferents, serotonergic afferents, rostral migratory stream

## Abstract

The present series of studies was designed to provide a general overview of the development of the region connecting the olfactory bulb to the forebrain. The olfactory peduncle (OP) contains several structures involved in processing odor information with the anterior olfactory nucleus (cortex) being the largest and most studied. Results indicate that considerable growth occurs in the peduncle from postnatal day (P)10–P20, with reduced expansion from P20 to P30. No evidence was found for the addition of new projection or interneurons during the postnatal period. GABAergic cells decreased in both number and density after P10. Glial populations exhibited different patterns of development, with astrocytes declining in density from P10 to P30, and both oligodendrocytes and microglia increasing through the interval. Myelination in the anterior commissure emerged between P11 and P14. Dense cholinergic innervation was observed at P10 and remained relatively stable through P30, while considerable maturation of serotonergic innervation occurred through the period. Unilateral naris occlusion from P1 to P30 resulted in about a 30% reduction in the size of the ipsilateral peduncle but few changes were observed on the contralateral side. The ipsilateral peduncle also exhibited higher densities of GAD67-containing interneurons and cholinergic fibers suggesting a delay in normal developmental pruning. Lower densities of interneurons expressing CCK, somatostatin, and NPY and in myelin basic protein staining were also observed. Understanding variations in developmental trajectories within the OP may be an important tool for unraveling the functions of the region.

## INTRODUCTION

**FIGURE 1:**
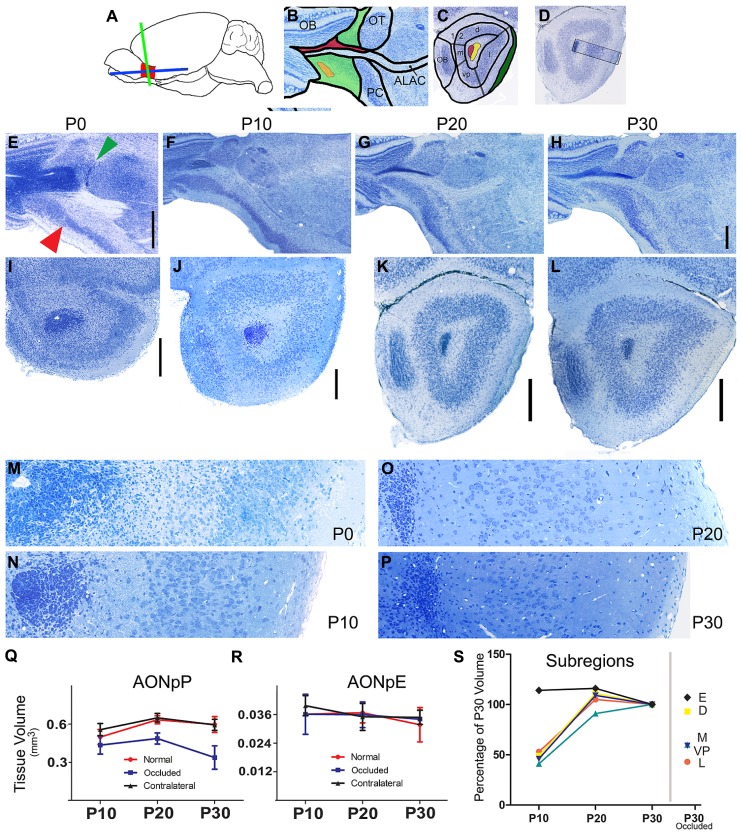
**Nissl-stained sections of the developing mouse olfactory peduncle. (A)** Diagram depicting the OP (red), and the two planes of section used in the subsequent panels: horizontal (blue) and coronal (green). **(B)** Diagram of regions seen in horizontal sections: rostral is to left and medial to top. OB, olfactory bulb; OT, olfactory tubercle; PC, piriform cortex; ALAC, anterior limb of the anterior commissure. Green, AONpP; orange, AONpE; red, subventricular zone/rostral migratory stream. **(C)** Diagram of regions seen in coronal sections through the caudal peduncle: Dorsal to top, lateral to right. 1, 2 = Layers 1 (plexiform) and 2 (cellular) regions of the AONpP. The region is also divisible into pars lateralis (l, under the LOT, shaded green), pars dorsalis (d), pars medialis (m), and pars ventroposterior (vp). Red, subventricular zone/rostral migratory stream; Yellow, ALAC. **(D)** Diagram depicting approximate location of the 350 μm-wide standardized region in the lateral OP seen in panels M, N, O, P, and in subsequent figures. **(E–L) **From left to right the columns depict tissue from newborn (P0), P10, P20, and P30 pups. The two rows contain developmental sequences in horizontal **(E–H)** and coronal **(I–L)** planes. The general organization of the peduncle is apparent at birth **(E)**. Note the obvious transition from the AONpP to the anterior piriform cortex (red arrow) and olfactory tubercle (green arrow). Compared to the older ages the darkly staining SVZ/RMS occupies a much great proportion of the center of the peduncle and core of the olfactory bulb. The ALAC, seen as a large white matter tract lateral to the RMS in F and G, is not as well developed at P0. Scale bar is 500 μm [**(B–D)** scaled in **(D)]**. **(I–L)** Coronal sections taken from the caudal peduncle. **(E,F)**: 2 μm plastic sections. **(G,H)**: 60 μm vibratome sections. The caudal-most remnant of the OB and the LOT are at the left and right borders, respectively. All subsequent coronal images maintain this orientation. At P0, the olfactory ventricle is still open in the core of the SVZ/RMS. With increasing age, the SVZ/RMS occupies a more compact region in the center of the peduncle and the encircling white matter becomes broader and more defined. The cellular zone of the AONpP, Layer 2, and the superficial plexiform layer (Layer 1) also become larger and more defined. Scale bars is 250 μm. **(M–P)**. 2 μm plastic sections taken from the SVZ/RMS (left) to the pial surface. With increasing age, the cell bodies in Layer 2 become less dense and the depth of Layer 1 increases. Each picture is 350 μm tall. **(Q,R)** Age-related changes in the total volume of the AONpP **(Q)** and AONpE **(R)**. In control animals (red) AONpP exhibited considerable growth from P10 to P20, and remained relatively stable from P20 to P30. Unilateral naris occlusion (blue) resulted in a decrease in the size of the ipsilateral peduncle but had little effect on the contralateral side (black; “contralateral”). AONpE exhibited fewer age-related changes and less of an effect of naris occlusion. **(S)** Left side: data depicting the growth of AONpP pars lateralis [orange, dorsalis (yellow) medialis (blue] and ventroposterior (light blue) and AONpE (blue) using P30 volume as 100% of size. The constituent layers of AONpP exhibited rapid change from P10 to P20 (yellow – pars dorsalis,D; red – pars lateralis, L, turquoise – pars ventroposterior, V; dark blue: pars medialis, M), while few age-related changes were seen in AONpE (black). The data points on the right side of the graph show compare the sizes of the various regions in pups with unilateral naris occlusion from P1 to P30 to the data at left from normal controls at P30. Pars lateralis **(L)**, ventroposterior (VP) and medialis **(M)** exhibited the greatest reduction in size while both pars dorsalis **(D)** and AONpE **(E)** exhibited relatively little change.

The olfactory peduncle (OP) connects the olfactory bulb (OB) to the remainder of the forebrain. All six neural structures found in the OP are involved in processing odor information. In mice, rats, and hamsters, the OB overlaps into the rostromedial peduncle, while the anterior piriform cortex (APC) and olfactory tubercle protrude into the caudolateral and caudomedial sides, respectively (**Figure 1**; Brunjes et al., 2005, 2011; Brunjes, 2013). Three areas are primarily contained within the OP (Brunjes et al., 2005, 2011). The largest is the anterior olfactory nucleus (AON; also referred to as the anterior olfactory cortex) which comprises two substructures: a large ring of cells known as the pars principalis (AONpP) and a small, superficial ribbon of neurons known as pars externa (AONpE). The AON receives direct input from the OB through axons in the lateral olfactory tract (LOT). It then provides feedback to the ipsilateral OB, feedforward information to the proximal dendrites of cells in the APC, and projects via the anterior limb of the anterior commissure (ALAC) to regulate the contralateral AON, OB, and APC. The two other structures, the ventral tenia tecta (VTT) and dorsal peducular cortex (DPC), extend into the region dorsal to the peduncle and have received relatively little attention (Haberly, 2001; Brunjes et al., 2005). The central core of the OP contains the subventricular zone with the “rostral migratory stream,” a pathway through which new neurons born in a proliferative zone at the edge of the anterior lateral ventricles migrate toward the OB (SVZ/RMS: Doetsch et al., 1997; Cummings et al., 1997a; Abrous et al., 2005; Imayoshi et al., 2009; Ming and Song, 2011).

Our recent studies have characterized a number of features of the OP in mature mice ranging from examinations of the major classes of projection and interneurons, the organization of the LOT and ALAC, and the centrifugal afferents coursing through the region (Brunjes et al., 2011; Brunjes, 2013; Kay and Brunjes, 2014). Few studies have examined the development of the OP and most have employed rats (Brunjes et al., 2005). The work has revealed a general, caudal-to-rostral gradient of neurogenesis in the OP during the last week of gestation, with several different regional patterns within the area (Bayer, 1986). For example, “transitional” zones (e.g., the VTT, DPC, and the junction between the AONpP and piriform cortex) exhibit an “inside-out” pattern of cell addition typical of the piriform cortex and neocortex. AONpP, which occupies the bulk of the OP, develops in the opposite fashion; from the outside-in. The AONpP’s distinct cell layer emerges by embryonic Day 19 and a substantially adult morphology is apparent by postnatal Day (P)15 (Schwob and Price, 1984). Brown and Brunjes (1990) reported variations in the growth of the major regions of AONpP between P10 and P60; such developmental variations suggest different architectures and therefore different functions.

The work described below was designed to provide a broad overview of developmental changes in populations of neurons (glutamatergic and GABAergic cells) and glia (astrocytes, oligodendrocytes, and microglia) as well as two neuromodulatory (cholinergic and serotonergic) systems that innervate the region. In addition, the work also provides a basic assessment of the effects of reducing input to the region by decreasing airflow through one side of the nasal cavity.

## MATERIALS AND METHODS

### ANIMALS

All procedures were performed according to NIH guidelines and protocols approved by the University of Virginia IACUC. All mice (C57Bl/6J Jackson Labs; Bar Harbor, ME, USA) were housed in standard polypropylene cages with food (8604, Harlan, Frederick, MD) and water *ad libitum*. The colony was maintained on a 12:12 light:dark cycle in a temperature- and humidity-controlled room. Data were collected from 3 to 8 pups for each measure on P10, P20, and P30 (the day of birth = P0). Studies of the development of inhibitory cells used hemizygous knock-in GAD67-GFP (Δneo) mice (C57BL6/J background) in which cDNA encoding enhanced GFP was inserted into the GAD67 locus, kindly provided by Dr. Yuchio Yanagawa (Gunma University Graduate School of Medicine; Tamamaki et al., 2003). Hemizygous GAD67-GFP males were mated with colony females. GFP-positive pups were identified at birth by visualizing the presence of fluorescent brain tissue with a dissecting microscope. Previous studies have indicated that GAD67-GFP mice have normal behavior and neuroanatomy (Tamamaki et al., 2003). To examine the role of normal experience in tissue development, some pups underwent unilateral naris occlusion (Coppola, 2012) on P1 and were examined at P30.

### NISSL STUDIES

Pups were deeply anesthetized with sodium pentobarbital (Euthasol, 0.39 mg drug/gm body weight; 150mg/kg) and perfused transcardially with 0.01 M phosphate buffered saline (pH 7.4; PBS) followed by 4% buffered formaldehyde freshly depolymerized from paraformaldehyde. The brains were removed and post-fixed for several days. One series of ages was embedded in glycol methacrylate (JB-4; Polysciences, Warrington PA), cut at 2 μm and stained with toluidine blue O; another, used to examine volumetric growth, was cut at 60 μm with a vibratome and stained with thionen. Changes in tissue size were made by estimating laminar volumes in serial sections and combining them using the Cavalieri principle.

### NEURONAL DEVELOPMENT

The development of pyramidal neurons was examined in sections double-immunostained with an antibody to Tbr-1 (a marker for glutamatergic neurons of pallial origin: Hevner et al., 2001; Englund et al., 2005; Ceci et al., 2012) and NeuN (which labels most CNS and PNS neurons: Mullen et al., 1992; **Table 1** contains antibody descriptions). Developmental changes in the number and distribution of GABAergic interneurons were examined in GAD67-GFP mice. Subpopulations of GABAergic cells that expressed one of three calcium-binding proteins (calretinin: CR, calbindin: CB, and parvalbumin: PV) or four peptide neurotransmitters (somatostatin: SOM, cholecystokinin: CCK, neuropeptide Y: NPY, or vasoactive intestinal polypeptide: VIP; Brunjes et al., 2011; Kay and Brunjes, 2014) were also examined. Potential postnatal neurogenesis was assessed by treating GAD67-GFP mice with a single injection of BrdU (50mg/kg; B5002 Sigma/Aldrich, St. Louis, MO; Magavi and Macklis, 2008) on P10, 20, or 30, harvesting the tissue 72 h later, and double-staining sections for Tbr-1 and BrdU.

**Table 1:** Antibodies.

Antigen	Immunogen	Manufacturer	Cat./lot #	Species	Dilution
5-HT	Serotonin coupled to BSA	Immunostar (Hudson, WI)	20080	Rabbit polyclonal	1/1000
			924005
BrdU	5-Bromo-2′-deoxyuridine	AbD Serotec (Kidlington, UK)	OBT0030CX	Rat monoclonal	1/200
			0412
Calbindin D-28k	Recombinant rat calbindin	Swant (Bellinzona, Switzerland)	300/ 07 (F)	Mouse monoclonal	1:1000
Calretinin	Rat calretinin	Millipore (Temecula, CA)	AB1550/2024183	Goat polyclonal	1:1000
CHAT	Human placental enzyme	Millipore (Temecula, CA)	AB144P	Goat polyclonal	1/100
			NG1780580
CCK	Gastrin-17	CURE Digestive Diseases Res. Center (Los Angeles, CA)	9303	Mouse monoclonal	1:1000
GFAP	Bovine spinal cord isolate	Dako, Carpinteria, CA	Z 0334	Rabbit polyclonal	1/500
			00076532
IBA-1	Synthetic peptide corresponding to the c terminus of IBA-1	Wako Richmond, VA	09-197421	Rabbit polyclonal	1/1000
			WEE4506
MBP	Human myelin basic protein	Millipore (Temecula, CA)	NE1019	Mouse monoclonal	1/500
			D00136729
NeuN	Purified mouse neuronal nuclei	Millipore (Temecula, CA)	MAB377	Mouse monoclonal	1/100
			2074765
NPY	Neuropeptide Y coupled to bovine thyroglobulin	ImmunoStar (Hudson, WI)	22940	Rabbit polyclonal	1:1000
			1112001
Parvalbumin	Parvalbumin purified from carp muscle	Swant (Bellinzona, Switzerland)	235	Mouse monoclonal	1:5000
			10(F)
Somatostatin	Somatostatin coupled to KLH	ImmunoStar (Hudson, WI)	20067	Rabbit polyclonal	1:5000
			216002
TBR-1	Amino acids 1–200 at the N-terminus of mouse TBR-1	Santa Cruz (Santa Cruz, Ca)	SC-48816	Rabbit polyclonal	1/100
			10409
VIP	VIP coupled to bovine thyroglobulin	ImmunoStar (Hudson, WI)	20077	Rabbit polyclonal	1:1000
			1129001

### GLIA AND NEUROMODULATORY INPUTS

An overview of the development of glia was undertaken by using immunofluorescence to visualize astrocytes (glial fibrillary acidic protein: GFAP), oligodendrocytes (myelin basic protein: MBP), and microglia (IBA-1; Ito et al., 1998). The myelination of the anterior commissure was also examined in pups taken from a single litter at P11, P12, P13, or P14. Developmental changes in the innervation of the OP by fibers from two neuromodulator systems (acetylcholine; CHAT and serotonin; 5-HT) were also examined.

### IMMUNOSTAINING AND ANALYSIS

Fluorescence immunohistochemistry was used to stain free-floating 60 μm-thick vibratome sections. Mice were perfused with freshly prepared paraformaldehyde as above and allowed to postfix for 2 h at 4^o^C. The sections were rinsed four times in 0.01 M PBS (pH 7.4). Next, the tissue was incubated in 0.01 M citrate buffer (pH 8.5) at 80°C (2 × 15 min, Jiao et al., 1999). After cooling at room temperature for 5 min, the sections were washed in PBS (2 × 2.5 min), permeabilized in 0.03% Triton in PBS (TW: 4 × 5 min), and placed into blocking solution (0.5% normal donkey serum in TW; Jackson ImmunoResearch, West Grove, PA) for 1 h. Sections were then placed into primary antibody (**Table 1**) at least overnight at 4^o^C. They were then washed (PBS 4 × 5 min) and incubated in secondary antibody (1/250 to 1/450 in TW: Jackson ImmunoResearch, donkey anti-rabbit: Catalog number, 711-165-152 or 711-545-152; donkey anti-goat: 705-165-147 or 705-545-147; donkey anti-mouse: 715-485-150 or 715-545-151; donkey anti-rat: 712-165-153) for 1 h, washed again (PBS 4 × 5 min) and mounted on slides with SlowFade mounting media (Invitrogen: S36937). To observe tissue organization, some sections were also Nissl-stained (640 nm Neurotrace; Invitrogen: N-21483). In each case, deletion of the primary antibody resulted in no staining.

Due to the large number of features examined (**Table 1**) and four developmental time points (P10, P20, P30, and P30 naris-occluded animals), we focused our quantitative analyses on two standardized regions. Most studies examined the same area of the caudal peduncle (the region where AONpP completely encircles the SVZ/ALAC core and Layer 2 is still separate from the overlying cerebral cortex, e.g., **Figures 1C, I–L**) used in previous work (Brunjes, 2013). In studies of GABAergic interneurons, anterior sections containing the entire lateral arc of AONpE were also selected. Images were collected with an Olympus confocal microscope. Montages were produced for each section by tiling images (10× for GABAergic interneurons, 20× for other images). For each image, either three (for GABAergic interneurons) or two optical sections separated by 3 μm were combined. Estimates of the number of labeled cells were accomplished by having two scorers tally labeled somata as well as measure the area of the targeted regions. Average number and densities were then calculated. Comparisons of the density of glia or centrifugal afferents were made from one 350 μm-wide column of tissue from the lateral side of the peduncle extending from the LOT to the ALAC (e.g., **Figures 1D, M–P**) from each subject. Image J (Rasband et al., 1997–2009) was used to transform (to 8-bit B&W) and threshold (using the “default” criteria) the images, and then to determine the percentage of foreground pixels in field (the “area fraction”; Salcedo et al., 2011). Images were acquired and minimally adjusted for brightness and contrast with Adobe Photoshop CS5 (San Jose, CA, USA) for the production of figures.

## RESULTS

### NISSL OVERVIEW

Although considerable maturation occurs during the early postnatal period, the basic organization of the peduncle is apparent at birth in the mouse. **Figures 1E–H** depict horizontal sections through the OP in P0, P10, P20, and P30 mice with the OB at left and the lateral portion of the peduncle at the bottom of each panel. Even at P0, there is a clear delineation between the AON and the more caudal PC: the cellular region of the AON is large and diffuse and thins rapidly into the PC at a fairly sharp border (**Figure 1E**, red arrow). An obvious boundary also exists on the caudomedial side between the AON and the OT (**Figure 1E**, green arrow). The SVZ/RMS is relatively large and takes up most of the core of the peduncle. The white matter region that surrounds the SVZ/RMS is very small, including the portion of it occupied by the ALAC (lateral to the SVZ/RMS).

**Figures 1I–L** depict coronal sections through the OP at roughly matching caudal locations. The left (medial) side contains the caudal remnant of the OB. The LOT (right) emerges as a well-defined bundle on the surface of the OP by P10. Once again, in P0 animals the SVZ/RMS is very large in the central peduncle and in some animals the olfactory ventricle is still patent. The delineation between cell poor Layer 1, cellular Layer 2, and the deep white matter over the SVZ/RMS is less apparent compared to later ages. **Figures 1M–P** contain images of 350 μm-wide strips of the lateral side of the OP extending from the SVZ/RMS to the pial surface. The increasing size of both the white matter on the superficial side (containing the LOT and Layer 1, at right of the figures) and the increased separation of the cell body layer (Layer 2) from the SVZ/RMS (left side) due to the expansion of the ALAC can easily be seen. The density of cells in Layer 2 decreased dramatically from P0 to P30.

Volumetric growth of the region was determined by measurements of serial sections in six animals at each age. Rapid expansion of AONpP occurred from P10 to P20, with total volume increasing by about one third (**Figure 1Q**, **Table 2**). During this time period, Layer 1 exhibited twice as much growth as Layer 2. Relatively little volumetric change was observed between P20 and P30. The regions that comprise AONpP (pars lateralis, dorsalis, medialis, and ventroposterior) exhibited similar patterns of growth, each attaining most of their P30 volume by P20 (**Figure 1S**). In contrast, AONpE size remained relatively constant from P10 to P30 (**Figures 1R,S**).

**Table 2:** Statistical results.

	P10	P20	P30	KW*	P1–P30 occluded	MW**
**Total volume (mm^3^)**						
AONpP	0.50 (8)	0.63 (6)	0.60 (8)	*H* = 13.39, *p* < 0.001	0.34 (4)	*U* = 4, *p* < 0.05
AONpE	0.04 (8)	0.04 (6)	0.03 (8)	*H* = 4.68, ns	0.03 (3)	*U* = 12, ns
**Numbers of GAD67-GFP-labeled somata in test sections**						
AONpP	1252 (5)	607 (3)	512 (4)	*H* = 8.79; *p* < 0.01	541 (3)	*U* = 0, *p* < 0.05
AONpE	108 (3)	83 (3)	71 (3)	*H* = 4.2, ns	74 (3)	*U* = 3, ns
**Percentage of test area exhibiting staining**						
GFAP	22.55 (8)	10.90 (4)	6.24 (4)	*H* = 9.72; *p* < 0.00	5.94 (3)	*U* = 5, ns
MBP	8.42 (4)	32.93 (4)	37.63 (4)	*H* = 7.73; *p* < 0.02	12.48 (5)	*U* = 1, *p* < 0.02
IBA-1	9.61 (4)	18.89 (4)	20.75 (4)	*H* = 7.73; *p* < 0.02	21.5(4)	*U* = 7, ns
5-HT	3.93 (6)	4.49 (4)	12.15 (4)	*H* = 5.81; *p* < 0.05	8.49 (4)	*U* = 5, ns
CHAT	16.18 (3)	11.41 (3)	13.01 (4)	*H* = 3.48, ns	26.04 (5)	*U* = 1, *p* < 0.05

Numbers, means (sample size); *, Kruskal–Wallis test of difference among control P10, P20, and P30 pups. **MW, Mann–Whitney test between control and P30 occluded animals. ns, not significant.

### NEURONAL DEVELOPMENT

**FIGURE 2:**
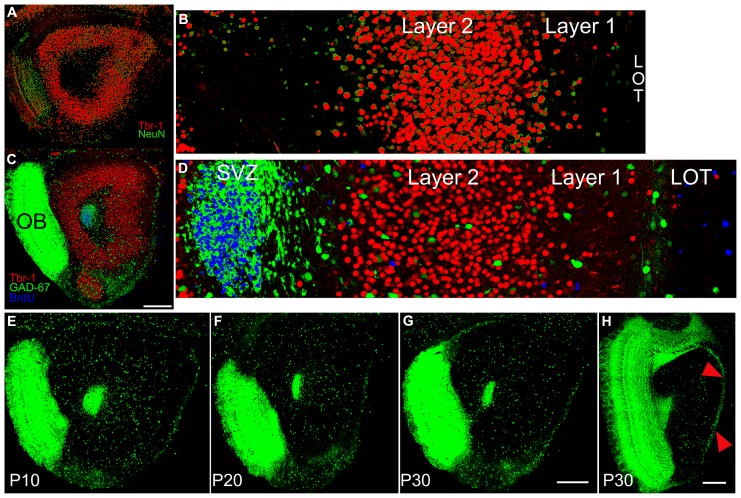
**(A)** Section from a P10 subject double stained for Tbr-1 (red) and NeuN (green) taken at the same level as the diagram in **Figure 1C**. **(B)** 350 μm-wide higher magnification view of the standardized region in the lateral OP. Tbr-1 immunoreactive cells fill Layer 2 of AONpP. Cells staining only for NeuN were found in large numbers in the granule cell layer of the OB (**A**, left side) scattered in both Layer 1 and the deep white matter of the core of the peduncle, and occasionally in Layer 2. **(C)** Section from a P10 GAD67-GFP pup stained for Tbr-1 and BrdU (injected 72 h previously). **(D) **350 μm-wide higher magnification view of the standardized region in the lateral OP. Blue BrdU labeling was heavy in the SVZ/RMS with cells also often observed along the pial surface. Only occasionally were stained profile observed in Layer 2 and most were not co-labeled as neurons. Scale bar in **A** and **C** is 300 μm. **(E–H)** Sections from GAD67-GFP mice at P10 **(E)**, P20 **(F)** and P30 **(G,H)**. To maximize the visibility of the sparse cells in the AON and provide consistent illumination through the bulk of the peduncle, the sections were overexposed. As a result, the densely packed, small GABAergic granule cells in the OB form a fused image. Note the decreasing number and density of GAD67-GFP cells in AONpP with increasing maturity **(E–F)**. In Panel **H**, the increased density of GAD67-GFP cells in AONpE (arrows) compared to the underlying AONpP is apparent (scale bars = 300 μm).

Tissue from P10, 20, and 30 pups was double immunostained for Tbr-1 (indicating glutamatergic neurons) and NeuN (a pan-neuronal marker) to assess general patterns of neuronal development. By P10, substantial numbers of cells immunostaining for both of the two markers were apparent in the AON, DPC, and VTT (**Figures 2A,B**). Relatively few single-labeled cells were observed in the peduncle except for the large population of OB granule cells staining only for NeuN. In the P10 tissue, about 60 single-labeled cells were observed per section in regions outside of the OB. Roughly 80% of these labeled for NeuN only. By P30, about twice as many single labeled cells were observed, with NeuN+ cells predominating by the same ratio. The prevalence of single-labeled cells was very small considering that the section illustrated in **Figure 2A** contains about 2000 Tbr-1 immunopositive cells. About 90% of the NeuN+/Tbr-1^-^ cells were found spread throughout Layer 1, at the superficial or deep margin of Layer 1, or in the ALAC; very few were found in the middle of Layer 2. There were no obvious patterns in the few Tbr-1+/NeuN- cells.

A second study examined whether new neurons were added to the region during the postnatal period. GAD67-GFP mice received a single injection of BrdU on P10, 20, or 30. The tissue was harvested 72 h later and stained for both Tbr-1 and BrdU (**Figures 2C,D**). We were not able to confirm previous reports (rats: Bayer, 1986; Frazier-Cierpial and Brunjes, 1989, mice: De Marchis et al., 2004) suggesting that at the ages examined here young neurons leave the rostral migratory stream and are incorporated into the AONpP. At all ages, large numbers of BrdU-labeled cells were found (a) within and scattered closely around the SVZ/RMS (b) within the OB granule cell layer, an expected finding given that the area is a main destination of the neuroblasts migrating through the RMS, and (c) within the LOT and along the pial surface, suggesting the addition of new glial cells. Occasional labeled cells were found in the ALAC and throughout the AONpP, TT and DPC in P10 and P20 pups, with fewer at P30. However, only very rarely were these cells co-labeled as either projection cells (Tbr1+) or interneurons (GAD67-GFP+). In sections containing nearly 2000 Tbr-1 figures and several hundred GAD67-GFP cells, perhaps one to four instances of co-labeling were observed. Such small numbers and the lack of discernable patterns strongly suggest that these results are artifacts of tissue preparation rather than evidence of neuronal addition.

**Figures 2E–H** contain images of tissue taken from GAD67-GFP mice at P10–P30. As expected, at all ages large numbers of fluorescent cells were observed in the SVZ/RMS and the region immediately around it, representing the migrating neuroblasts in the RMS. A dense collection of small GFP+ cells and processes was also observed in the OP ventral and medial to the LOT. These cells did not appear to be a portion of the VTT, which could be seen more medially as an obvious, circular region of cell bodies. At all ages, GAD67-GFP cells were observed in a loose line just deep to the LOT, though by P30 these cells were much less dense. In the AONpP total numbers of GAD67-GFP cells decreased dramatically with age: from P10 to P20 the number/section was reduced by half, and a further 15% drop occurred between P20 and P30 (**Table 2**). The loss in numbers, coupled with age-related growth of the region (**Figure 1Q**), resulted in large decreases in the density of interneurons, with values for P30 pups one third of those of the P10 subjects (means = 2.7 vs 7.4 cells/100 μm^2^).

A slightly different pattern was observed for AONpE (**Figure 2H**). While the density of GAD67-GFP cells in the region decreased from P10 to P30, the differences between ages were not significant (**Table 2**). Labeled cells were almost three times more dense in AONpE than in AONpP at P30 (mean = 7.7 vs 2.7 cells/100 μm^2^).

**FIGURE 3:**
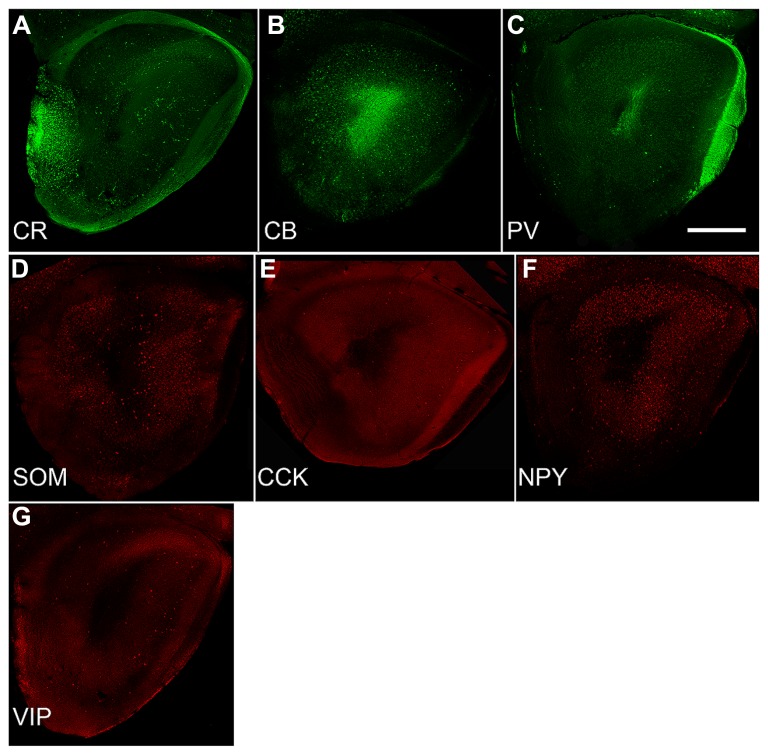
**Sections from P30 pups from a standardized location in the caudal OP (see **Figure 1C**) immunostained for the three calcium-binding proteins (**A**: calretinin; CR, **B**: calbindin; CB, **C**: parvalbumin; PV) and four peptides (**D**: somatostatin; SOM, **E**: cholescytokinin; CCK, **F**: neuropeptide Y; NPY, **G**: vasoactive intestinal polypeptide; VIP).** Note the wide differences in density and number. Comparisons with **Figure 2G** demonstrate that these seven varieties account for only a small proportion of the total population of GABAergic neurons (Kay and Brunjes, 2014). Scale bars = 300 μm.

Developmental changes in the numbers of cells that expressed one of three calcium-binding proteins (CR, CB, or PV) or four peptides (SOM, CCK, NPY, or VIP) were also examined (**Figure 3**). In the AONpP, the number of cells/section labeling for CB, PV, SOM, CCK, NPY, and VIP increased from P10 to P20 and decreased for CR (*U*’s –0, *p* < 0.05). The greatest increases occurred in pars lateralis. CCK cells were absent at P10. By P30, the seven different antigens could be divided into three different categories: dense (CB and CR; about 1000–1700 cells/mm^2^), moderate (NPY and VIP; 250–360 cells/mm^2^), and sparse (SOM, PV, and CCK; 70–130 cells/mm^2^). Observations indicated that: (a) at each age, highest numbers of immunopositive cells were observed in Layer 2 of AONpP except for CR, which was evenly distributed between Layers 1 and 2 (b) Examinations at all three ages the density of CB immunoreactive soma was lowest in pars ventroposterior, (c) CR levels were variable across ages, although generally highest in pars medialis and ventroposterior, (d) highest levels of VIP staining were observed in pars lateralis and dorsalis in P20 and P30 pups and highest levels of PV were observed in pars lateralis and dorsalis, SOM in pars ventroposterior and CCK in pars dorsalis. Examinations of AONpE at P30 revealed significant increases in the number of cells labeled for CB and VIP between P10 and P20 (*U*’s –0, *p* < 0.05). In AONpE the seven antigens could be grouped into two categories: relatively dense (CB and CR : 5000–14000 cells/mm^2^ ) and very light (VIP, NPY, COM, CCK, and PV, <300 cells/mm^2^).

### GLIAL DEVELOPMENT

**FIGURE 4:**
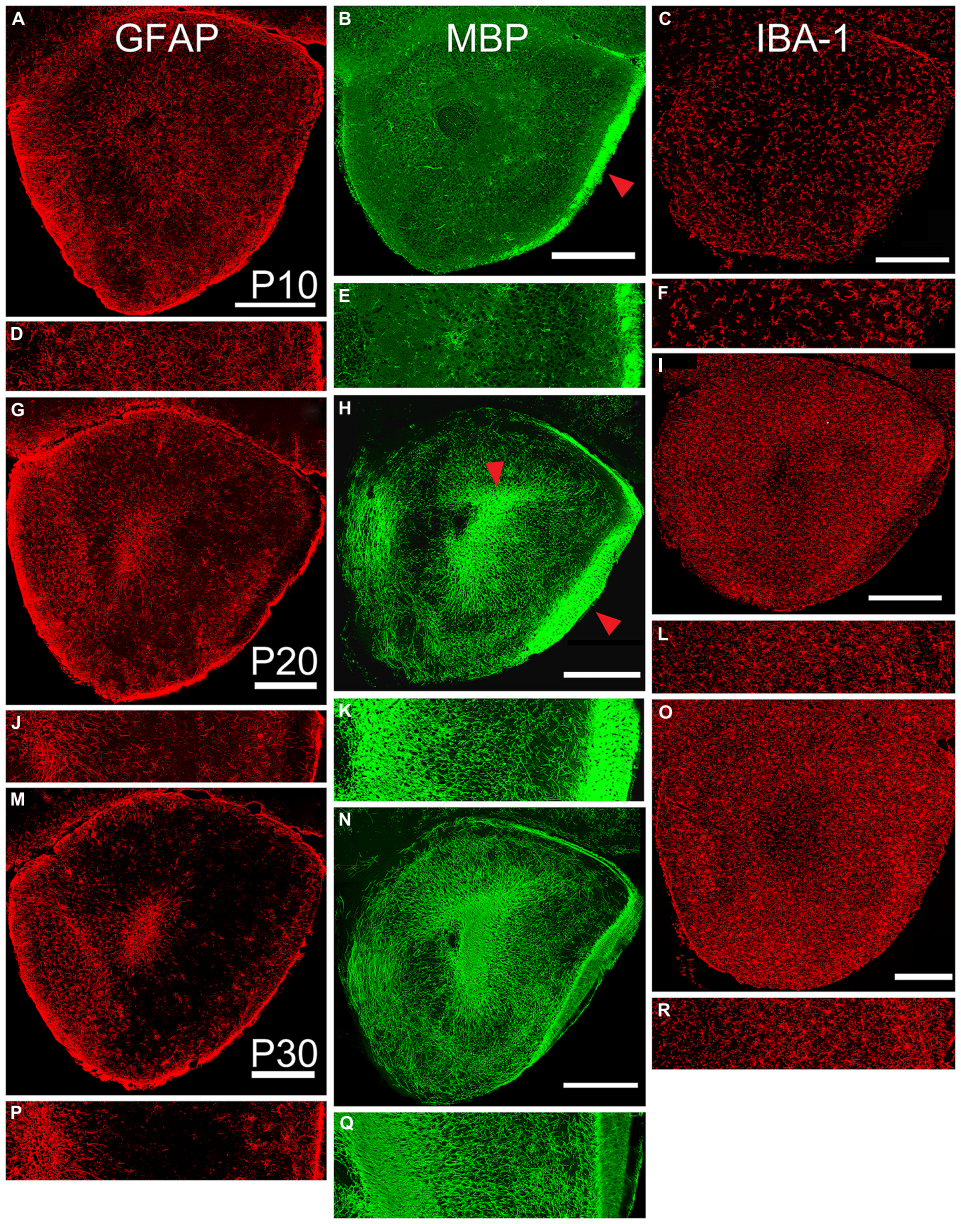
**Age-related changes in expression of markers for astrocytes (left column, GFAP), oligodendrocytes (middle column, MBP), and microglia (right column, IBA-1).** Paired low-power coronal images (taken at the same level as the diagram in **Figure 1C**) and 350 μm lateral strips in each column represent P10 (**A,D; B,E**; and **C,F**), P20 (**G,J; H,K,** and **I,L**), and P30 (**M,P**; **NQ**; and **O,R**). Scale bars on low-power images = 500 μm. While the density of staining for astrocytes exhibited an age-related decrease, the other two markers increased in expression during the age range examined. Red arrow in Panel **B** = LOT, Panel **H** = LOT and ALAC.

Astrocytic (GFAP immunoreactive, **Figure 4**, left column) cells and processes filled the peduncle at P10 with remnants of radial glial processes extending from the core to the pial surface. At all ages, staining was heavy in the SVZ/RMS (representing the glia that support chain migration of neurons in the RMS, Doetsch et al., 1997; Ghasheghaei et al., 2007), in the granule cell layer of the OB, and near the pial surface and LOT. Staining in Layer 2 progressively diminished with age. This was reflected by a 50% decrease in the density of staining in the lateral test zone from P10 to P20 and another 50% decrease from P20 to P30 (**Table 2**).

**FIGURE 5:**
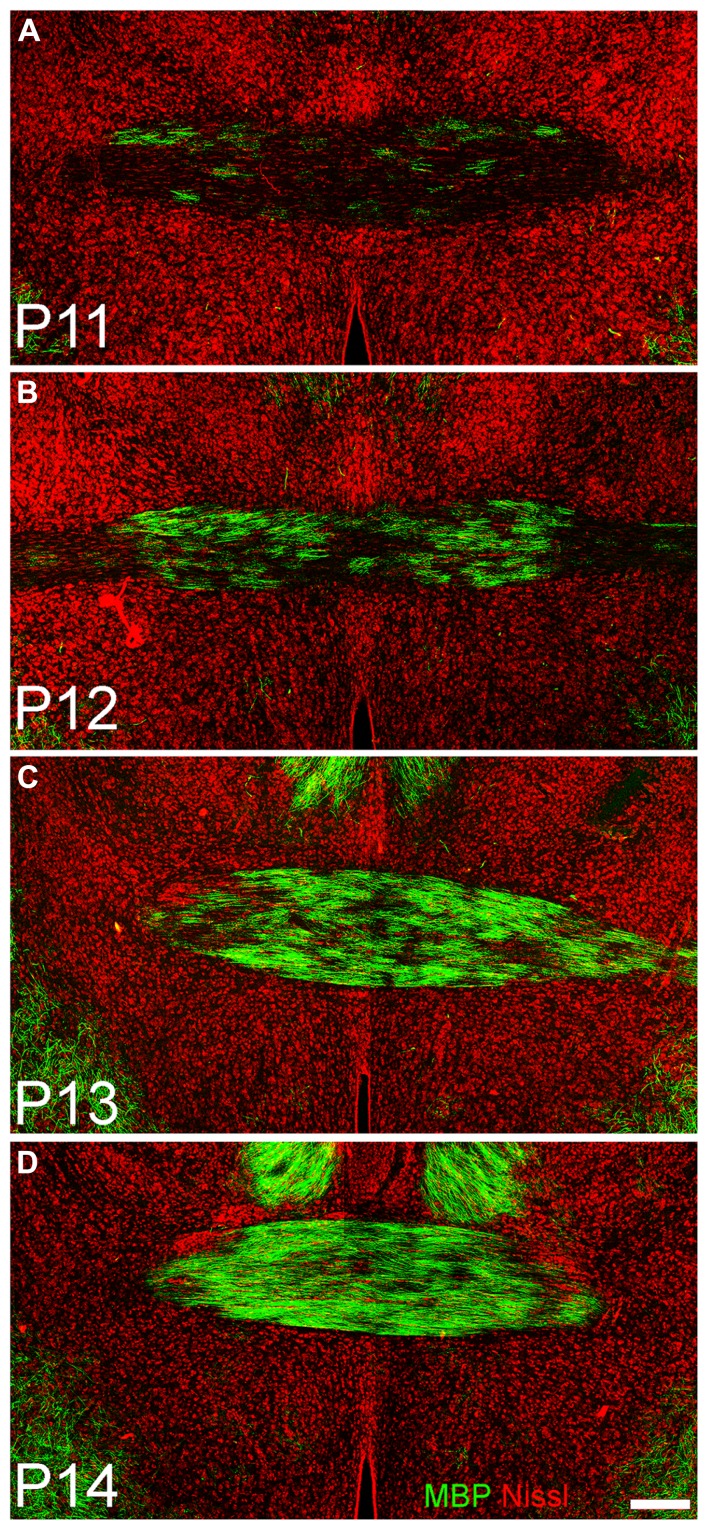
**Coronal sections taken through the anterior commissure at P11 (A), P12 (B), P13 (C), and P14 (D).** Dorsal to top. A few fibers expressing myelin basic protein (green) can be seen traversing horizontally in the commissure at P11; by 72 h later **(D)** the region is densely filled. Red = Nissl stain. Scale bar = 200 μm.

Immunostaining for oligodendrocytes (MBP, **Figure 3**, middle column) was light and variable at P10. Only one of the two large myelinated tracts in the region, the LOT (arrow **Figure 4B**), exhibited consistent staining. By P20, bright immunofluorescence was observed in both the LOT and ALAC (arrow, **Figure 4H**). Measures taken from the lateral test zone revealed a tripling in the density of stained fibers from P10 to P20 with an additional 10% increase from P20 to P30 (**Table 2**). Since the ALAC carries axons, several millimeters caudal of the peduncle where they cross to the contralateral hemisphere through the AC, a more detailed examination was performed with tissue taken from littermates at 24-h intervals (**Figure 5**). Small bundles of MBP-positive fibers were observed extending through the AC at P11 and P12. By P14, the AC was filled with staining. In both P20 and P30 samples, the deep white matter region separating Layer 2 of AONpP from the SVZ/RMS was obvious, thickly encircling all but the medial side of the SVZ/RMS. The relative lack of staining on the medial side of the SVZ/RMS is because this portion of the AON, pars medialis, is the only region without a substantial contralateral projection (Brunjes et al., 2005).

The entire OP was filled with microglia (IBA-1, **Figure 4**, right column) from P10 to P30. The distribution was uniform with a slight increase in density under the LOT and reduced numbers within the SVZ/RMS. With age, the density of the staining increased (**Table 2**). The density of stained profiles in the lateral test zone doubled between P10 and P20 and increased only slightly from P20 to P30.

### NEUROMODULATORY INPUTS

**FIGURE 6:**
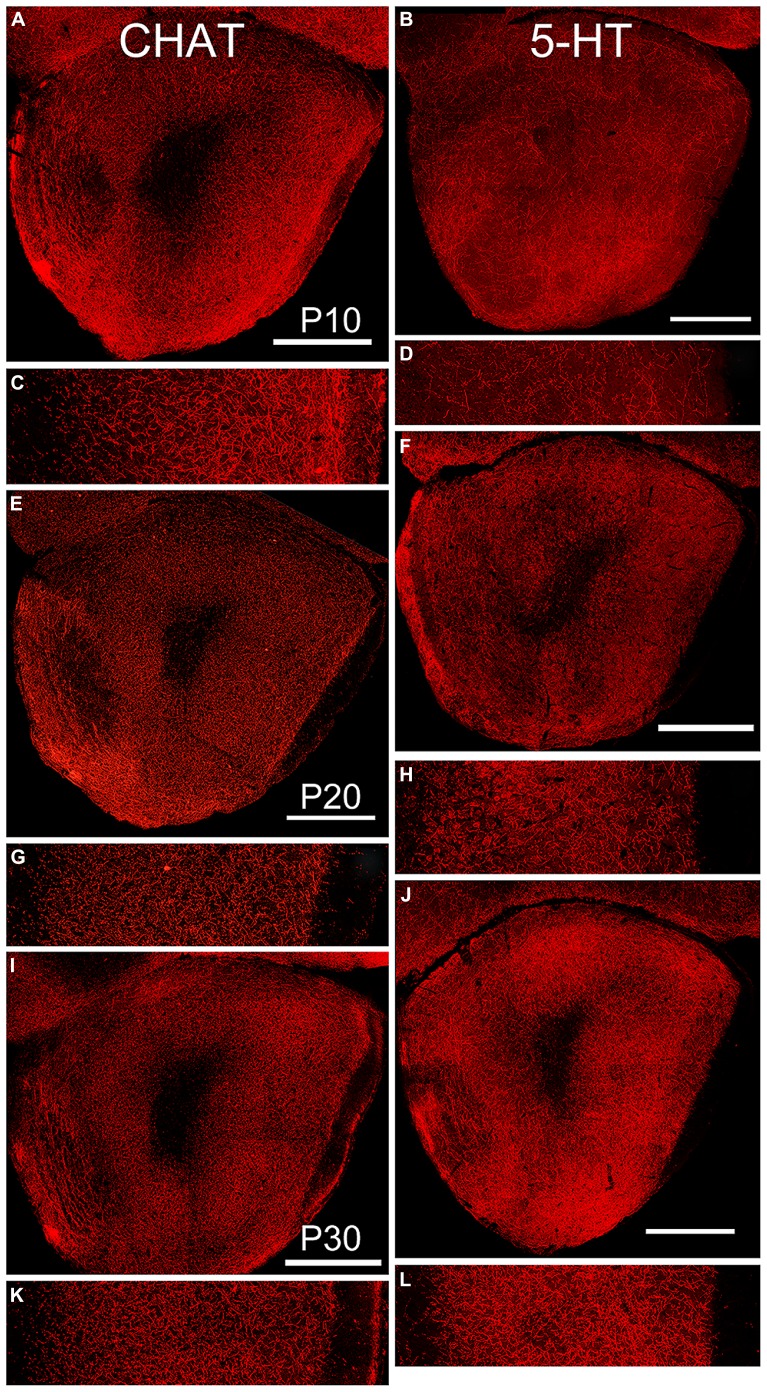
**Age-related changes in expression of markers for cholinergic (left column, CHAT), and serotonergic (right column, 5-HT) fibers.** Paired low-power coronal images (taken at the same level as the diagram in **Figure 1C**) and 350 μm lateral strips in each column represent P10 **(A,C** and **B,D)**, P20 **(E,G** and **F,H)** and P30 **(I,K** and **J,M)**. Scale bars on low-power images = 500 μm. At P10, fibers of both antigens filled the OP. With age, the density of cholinergic fibers decreased while the density of serotonergic fibers increased. Somatic labeling with CHAT was often observed.

By P10, the basic pattern of cholinergic innervation of the OP was apparent (**Figure 6**, left column). A complex meshwork of immunostained fibers filled Layers 1 and 2 of AONpP, while the deep white matter of the peduncle and LOT was mostly unstained. Immunostaining was heavy in Layer 1 just deep to the LOT but less dense nearer the cell body layer. Layer 2 was heavily labeled with no apparent regional differences. At the dorsal superficial edge of the peduncle, labeled fibers were often visible extending circumferentially. Labeled cell bodies were observed in nearly every section (~3–15/section) and, though widespread, were most likely to be encountered in pars lateralis. While the density of staining in the lateral test area was higher in P10 pups, no statistical differences were observed across ages (**Table 2**).

By P10, substantial numbers of 5-HT fibers were also apparent throughout the OP (**Figure 6**, right column) though relatively scarce in both the ALAC and LOT. Few regional differences were observed consistently across subjects except for a high fiber density in the dorsomedial region where the OP connects with the frontal neocortex, and low densities in the region of the VTT-containing cell bodies. A different developmental pattern emerged than that observed for CHAT. The density of 5-HT fibers was similar in P10 and P20 pups, but doubled between P20 and P30 (**Table 2**). As reported previously (Brunjes, 2013), the density of 5-HT axons was much lower than that of cholinergic fibers, and all olfactory glomeruli were heavily labeled.

### EFFECTS OF UNILATERAL NARIS OCCLUSION

Blocking airflow through one half of the nasal cavity resulted in an approximate 12% reduction in the size of the ipsilateral AONpP by P20 and by P30 the region was about one third smaller than control values (**Figure 1Q**, **Table 2**). Greatest reductions in volume occurred in AONpP pars lateralis, medialis, and ventroposterior (**Figure 1S**, right). The plexiform Layer 1 exhibited larger reductions in size than cellular Layer 2. Tissue from the side contralateral to occlusion in experimental mice did not differ in size from normal controls and the procedure did not appear to affect AONpE (**Figure 1R**).

Since the well-documented decrease in the size of the OB is primarily due to cell death in interneuron populations (Fiske and Brunjes, 2000; Coppola, 2012) and the results above indicate that in normal pups there is an age-related decrease in the density of GABAergic cells from P1 to P30, it seemed possible that changes in populations of GAD67-GFP cells might contribute to the observed differences in tissue volume. However, tissue from pups occluded from P1 to P30 actually revealed higher numbers of GABAergic cells on both the occluded and contralateral sides than those in controls (**Table 2**). In fact, the numbers were similar to those observed for normal pups at P20. Attempts to analyze whether naris occlusion resulted in changes in the number of cells exhibiting one of the seven GABAergic subpopulations in P10 and P20 pups were confounded by the relatively small numbers of cells that expressed the markers and thus the high variability between sections. By P30, numbers of cells labeled for SOM, CCK, and NPY in AONpP were lower in occluded tissue than in normal controls (*U*’s = 0; *p* < 0.05). No differences were observed in AONpE.

The effects of naris occlusion on the centrifugal afferents and glia populations were assessed by measuring the density of stained profiles in the 350 μm-wide lateral strips of the caudal peduncle described above. Due to the small sample sizes, no consistent differences could be found between the tissue ipsilateral and contralateral to occlusion in experimental animals. Furthermore, no differences in serotonergic, GFAP or IBA1 staining were observed between tissue from the occluded side and samples obtained from normal control animals. However, the density of staining for CHAT and MBP (**Table 2**) was consistently higher on the occluded side than on the contralateral.

## DISCUSSION

The present study is the first thorough examination of the postnatal development of the mouse OP. All component areas of the region are recognizable on the day of birth, but considerable maturation takes place in the first 20 postnatal days. Layer 1 exhibited the largest amount of expansion during early life; as a plexiform layer the results indicate that considerable synaptogenesis must occur in the region during this period.

The SVZ/RMS, a major migratory pathway for young neurons, extends from the anterior lateral ventricles through the core of the OP and into the OB (**Figure 1**; Brunjes et al., 2005; Brunjes, 2013). The system also provides neurons to both cortical and subcortical structures through a “ventral migratory mass,” providing cells to nucleus accumbens and olfactory tubercle, and a “ventrocaudal migratory stream” traveling along the border between n. accumbens and the AON to the olfactory tubercle (De Marchis et al., 2004). At birth, the SVZ/RMS commands a large percentage of the core of the OP (**Figures 1E,I**), an expected finding given that it supplies over 90% of the OB’s population of interneurons in the first postnatal week (Hinds, 1968; Bayer, 1983). It has been reported that in young mice (P1–P6) the SVZ/RMS supplies new neuron for structures in the OP based on observations of scattered NeuN-labeled cells in the area surrounding the main body of migrating cells (De Marchis et al., 2004; see also Bayer, 1986). The results presented above indicate that in P10, P20, and P30 mice BrdU-labeled cells do not co-localize either with Tbr-1, a marker of glutamatergic projection neurons (Hevner et al., 2001; Englund et al., 2005; Ceci et al., 2012), or GAD-67, which identifies nearly all GABAergic cells in the region (Kay and Brunjes, 2014), suggesting that any new cells at these ages are likely to be glia. The SVZ/RMS thins considerably by P30, by which time is it encircled by a broad white matter region that includes the ALAC (**Figures 1H,L**).

A dramatic reduction in both the number and density of cells expressing GAD67 was observed from P10 to P30. The result is consistent with the developmental pruning of interneurons that occurs during early postnatal life in other regions, including the OB (e.g., Fiske and Brunjes, 2001; Hoopfer et al., 2006; Salcedo et al., 2011). The large increases in the number of interneurons expressing CB, PV, SOM, CCK, NPY, and VIP observed between P10 and P20 suggest that during this period many cells develop mature neurophenotypes.

The development of glia was examined by visualizing astrocytes (GFAP), oligodendrocytes (MBP), and microglia (IBA-1). Different patterns were observed for each marker. Astrocytes densely filled the peduncle on P10, but by P30 much less staining was observed, with high levels only in the SVZ/RMS (where they form the substrate for neuroblast migration to the OB; Doetsch et al., 1997), and in superficial regions. In contrast, oligodendrocytes and myelin were scarce at P10: an occasional profile could be observed in Layer 2 and the LOT exhibited variable staining. By P20, immunostaining had intensified throughout the peduncle and the ALAC was apparent (**Figure 4H**). The ALAC contains axons from the contralateral AON that cross in the anterior commissure (Brunjes, 2013). Examination of the commissure at sequential ages indicated that only a few myelinated fibers were apparent at P11 but that the region was filled 72 h later (**Figure 5**). While contralateral projections have been reported to exist from birth (Math and Davrainville, 1980), the bulk of the commissure develops around P12 in the rat (Schwob and Price, 1984). Interestingly, the AC disappears with the elimination of OB mitral cells in PCD mice suggesting that its development is under complex, transynaptic regulation (Recio et al., 2007). The functional maturation of the commissure has been demonstrated in studies in which pups are unilaterally conditioned with a novel odor. Odors presented to the contralateral side are not recognized until about P12 (Kucharski and Hall, 1987, 1988; Fontaine et al., 2013). Other evidence suggesting that the OP may be involved in the bilateral processing of learned information include reports that learning enhances 2-deoxyglucose uptake in the AONpP pars dorsalis of trained rats compared to controls (Hamrick et al., 1993) and enhanced fos expression in the AON following aversive odor conditioning (Funk and Amir, 2000). Finally, as has been reported in the OB (Caggiano and Brunjes, 1993; Fiske and Brunjes, 2000), microglia (IBA-1) staining was plentiful in P10 animals and cells became even denser in the older ages.

Two centrifugal afferents were also examined and found to have different developmental histories. Cholinergic fibers have been reported to densely fill the peduncle in adult mice (Durand et al., 1998; Brunjes et al., 2011), and the same proved true by P10. Levels remained high throughout the first four postnatal weeks. Salcedo et al. (2011) found a different pattern of development in the mouse OB: a large increase in fiber density from P2 to P12 followed by a decrease from P12 to adulthood. There are several reasons why the two regions might show different patterns. First, the OB has a very different developmental history, with the large numbers of new neurons added during the first postnatal week resulting in a very large expansion of tissue volume. Second, all cholinergic fibers entering the bulb must travel through the OP; their earlier residence in the structure affords more time to mature. Interestingly, a few labeled cell bodies were found in AONpP in every subject, suggesting a small constituent population of cholinergic neurons. Serotonergic innervation occurred with a very different time course. While present at P10, fiber density tripled between P20 and P30. As in the adult (Brunjes, 2013), 5-HT fibers were not as dense as CHAT staining.

Many studies have demonstrated that unilateral naris occlusion near birth results in about a 25% decrease in the size of the ipsilateral OB by P30 (Coppola, 2012). The effects of the procedure on the development of the AON are much less clear, but perhaps more interesting given that the AON is a major locus of crossed projection in the olfactory forebrain. Naris occlusion does affect the function of the AON as metabolic markers are reduced in the region compared to controls (Brown and Brunjes, 1990). Comparing the present results with previous work is difficult for two reasons: (a) most published work has been done in rats and (b) different investigators employ varying durations of occlusion. The work reported above indicates that closure from P1to P30 in mice reduces the size of the ipsilateral AONpP by about one third with little effect on the contralateral side and little effect on AONpE. Occlusion for the same period has been reported to not affect laminar growth on either side in rats (Brown and Brunjes, 1990). Substantial species differences in the effects of naris occlusion have been reported before (e.g., Brunjes, 1988; Cummings et al., 1997b), perhaps explaining the differences between rats and mice. Barbado et al. (2001, 2002) reported that much more extended periods of occlusion (P1–P60) in rats results in large reductions in AONpP volume on the ipsilateral side along with smaller changes on the contralateral side. Furthermore, regional differences were noticed, with occlusion having more effects in the anterior vs posterior AONpP, and were most pronounced in pars lateralis and AONpE (Barbado et al., 2001). Barbado et al. (2002) also examined CB, CR, and PV immunoreactive cell density and reported alterations on both the ipsilateral and contralateral sides. Their studies were important in that they were among the first to suggest contralateral effect of naris occlusion.

We found several other effects in pups occluded from P1 to P30. First, numbers of GAD67-GFP-positive interneurons were higher in occluded animals, while the density of GABAergic neuron subspecies expressing the peptides CCK, SOM, and NPY decreased. The density of staining for CHAT and MBP was consistently higher on the occluded side than on the contralateral. While the exact reasons for these changes remain to be explored in detail, one plausible explanation for the high density of GABAergic cells and CHAT staining is that the reduction in activation that occurs with occlusion results in a delay in normal developmental pruning (Hoopfer et al., 2006; Salcedo et al., 2011). The values observed for tissue from the experimental side in occluded animals at P30 resembled those seen in normal pups 10 days younger.

Relatively little is known about the structure and function of the regions that comprise the OP. The studies reported above provide a general summary of patterns of development across several different measures and examine the role of afferent input as a factor in early growth. Considerable differences were observed, from relatively linear growth to substantial declines. Continued research will be needed to understand the regional differences in structure and function that might account for these developmental trajectories.

## Conflict of Interest Statement

The authors declare that the research was conducted in the absence of any commercial or financial relationships that could be construed as a potential conflict of interest.
